# *Allium Macrostemon* Bge. Attenuates the Cognitive Decline of Aging Mice by Enhancing BDNF/TrkB Pathway

**DOI:** 10.1002/fsn3.70010

**Published:** 2025-02-28

**Authors:** Ruilin Sheng, Meihuan Zhao, Keting Pu, Yongtao Zhou, Li Zeng, Yuanyuan Chen, Ping Wang, Xiao Liu, Shijun Xu

**Affiliations:** ^1^ State Key Laboratory of Southwestern Chinese Medicine Resources Chengdu University of Traditional Chinese Medicine Chengdu China; ^2^ School of Pharmacy Chengdu University of Traditional Chinese Medicine Chengdu China; ^3^ Institute of Material Medica Integration and Transformation for Brain Disorders Chengdu University of Traditional Chinese Medicine Chengdu China

**Keywords:** aging, *Allium macrostemon* Bge, BDNF/TrkB pathway, cognitive impairment, synaptic plasticity

## Abstract

*Allium macrostemon* Bge. (AM) is a widely utilized culinary spice recognized for its numerous health‐promoting properties. Aging‐related cognitive impairment (ARCI) represents a significant global health concern during the aging process. However, the potential of AM to attenuate ARCI has not been investigated. This work aims to reveal the effects and potential mechanisms of the water extraction of AM (WEAM) in alleviating ARCI, with a particular emphasis on the BDNF/TrkB signaling pathway. The findings showed a significant enhancement in memory function and a reduction in hippocampal neuronal damage in aging mice following treatment with WEAM, manifested by an increased spontaneous alternation rate in the Y‐maze, prolonged step‐through latency, and decreased number of errors in the PAT test, a shortened escape latency and increased platform swimming time and platform crossing times in the MWM test. Additionally, WEAM reduced oxidative stress, elevated the expression of proteins related to synaptic plasticity (SYN and PSD95), and activated the BDNF/TrkB signaling pathway in D‐galactose‐induced aging mice. To elucidate the mechanism by which WEAM alleviates ARCI, both a TrkB activator (7,8‐DHF) and an inhibitor (ANA‐12) were employed. The results demonstrated that the effects of WEAM on synaptic plasticity were potentiated by 7,8‐DHF and diminished by ANA‐12. Finally, 11 chemical compositions of WEAM were analyzed and quantified using HPLC‐MS/MS, including macrostemonoside, sarsasapogenin, diosgenin, timosaponin AIII, N‐p‐trans‐coumaroyltyramine, guanosine, adenosine, phenylalanine, adenine, arginine, and valine. These results suggest that AM may serve as a promising culinary spice for mitigating ARCI by promoting the BDNF/TrkB signaling pathway, thereby enhancing synaptic plasticity.

## Graphical abstract

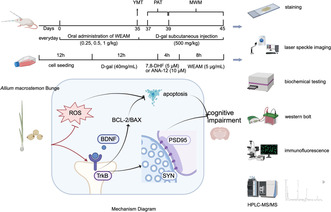

**Abbreviations**
7,8‐DHF: 7,8‐Dihydroxyflavone
AM: *Allium macrostemon* Bge.
ARCI: Aging‐related cognitive impairment
BAX: BCL2‐Associated X
BCL‐2: B‐cell lymphoma‐2
BDNF: Brain‐derived neurotrophic factor
D‐gal: D‐galactose
DMEM: Dulbecco's Modified Eagle's Medium
HE: Hematoxylin and eosin
MDA: Malondialdehyde
MEM: Memantine
MWM: Morris water maze
PAT: passive avoidance test
PSD95: Postsynaptic density‐95
ROS: Reactive oxygen species
SOD: Superoxide dismutase
SYN: Synaptophysin
TdT: Terminal deoxynucleotidyl transferase
TrkB: Tyrosine kinase B
TUNEL: dUTP nick‐end labeling
WEAM: water extraction of Allium macrostemon Bge.
YMT: Y‐maze test

## 1 Introduction

Aging contributes to human cognitive decline (Nyberg and Pudas 2019), and one in six individuals worldwide will be 60 years or older by 2050 (World Health Organization 2022). Aging‐related cognitive impairment (ARCI) is a neurodegenerative condition that is closely linked to the deterioration of synaptic plasticity and has become a pressing global health issue as populations age (Nyberg and Pudas 2019; He et al. 2024). An expanding body of research highlights the essential influence of dietary factors on cognitive performance (Dalile et al. 2022; Ellouze et al. 2023). Several edible plants, such as ginseng, *zanthoxylum bungeagum* maxim., and ginkgo, have been shown to exert positive effects on ARCI (Barbalho et al. 2022; Zhang, Chen, et al. 2022; Zhang, Ni, et al. 2022; Zhao et al. 2021). Therefore, dietary interventions, particularly those incorporating bioactive compounds with medicinal properties, may offer a viable strategy for maintaining cognitive health.

Excessive oxidative stress, neuronal apoptosis, and impairments in synaptic plasticity are the pathological hallmarks of ARCI (Plascencia‐Villa and Perry 2023; Verdú et al. 2023; Kalinichenko et al. 2023). The BDNF/TrkB neurotrophic signaling, involved in neuronal development, differentiation, and survival, defects are closely related to neurodegenerative changes (Wang et al. 2019; Kuo, Lin, and Lane 2021; Numakawa and Odaka 2022). Maintaining proper BDNF/TrkB signaling in the hippocampus is crucial to hippocampal phenotype and spatial memory in obese mice under psychosocial stress (Agrimi et al. 2019). The decreased blood circulation to the heart and reduced levels of BDNF in the brain are closely associated with physiological aging (Elia et al. 2021). Interestingly, activated BDNF/TrkB signaling pathway can counteract cognitive decline, whether through BNDF supplementation or stimulation of TrkB with agonists (Nagahara et al. 2009; Zagrebelsky and Korte 2023; Sun et al. 2023). Thus, enhancing the BDNF/TrkB pathway may represent an effective strategy for relieving ARCI (Zhang et al. 2021).

**FIGURE 1:**
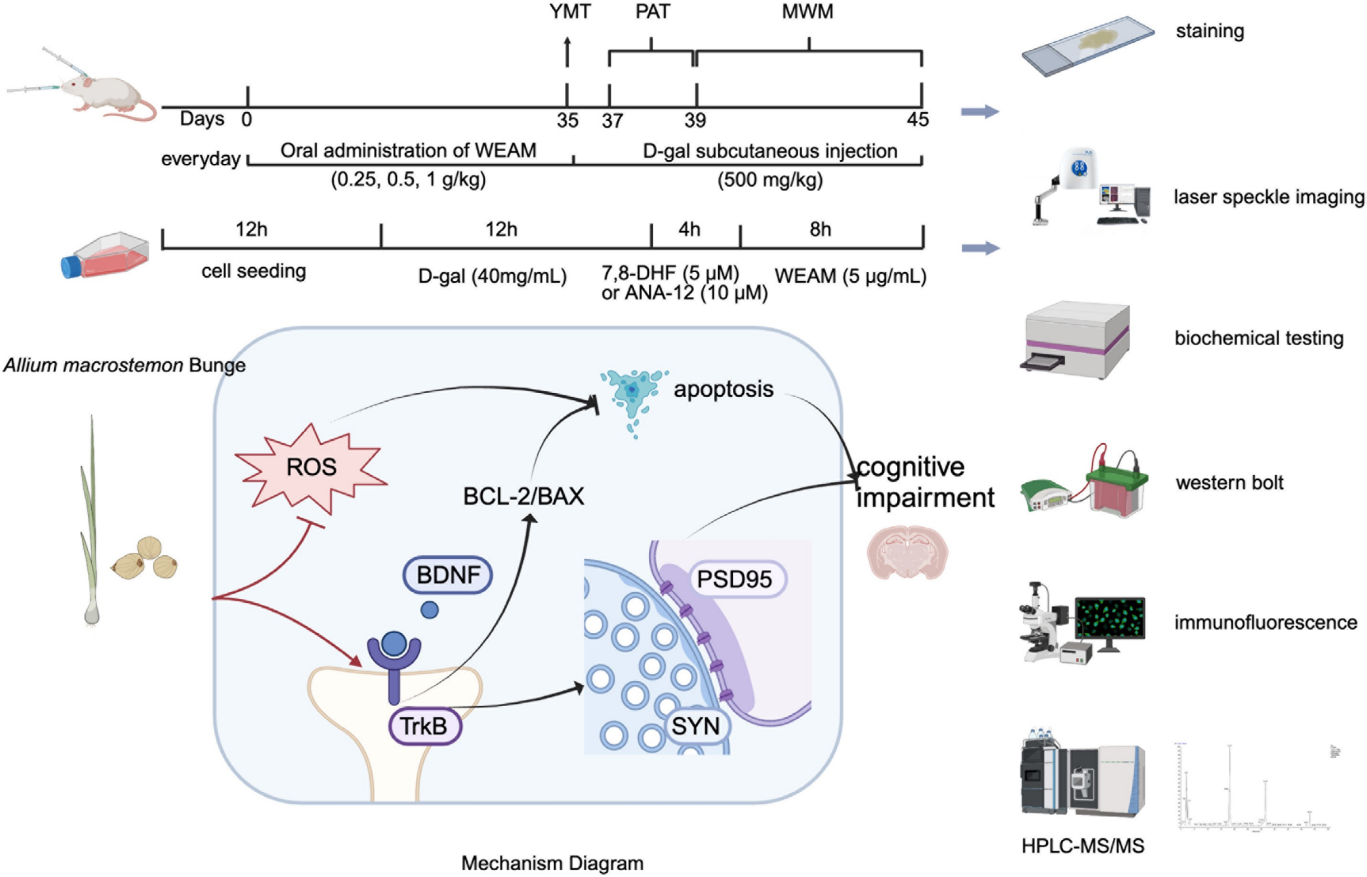
Graphical abstract.

*Allium macrostemon* Bge., (AM) is a well‐known culinary spice, commonly referred to as Jiaotou, and is widely used in Southeast Asia and Russia (Qin et al. 2023). Traditionally, AM has been applied to alleviate conditions such as thoracic pain, stenocardia, and amyocardia for thousands of years (Lin et al. 2024), which is attributed to its antioxidant, anti‐inflammatory, anti‐atherosclerosis, and antihyperlipidemic properties (Yang et al. 2021; Qin et al. 2023; Wu et al. 2023, 2020). Moreover, research has demonstrated that the administration of AM can elevate the levels of BDNF in the brains of depressed mice (Lee et al. 2010). However, it is an open question whether AM can relieve ARCI by facilitating the BDNF/TrkB signaling pathway. D‐galactose (D‐gal) is recognized for its ability to induce excessive ROS production ROS, resulting in neuronal injury, and is widely utilized as an experimental model for physiological aging (Azman and Zakaria 2019; Hakimizadeh et al. 2021). This study aims to investigate the effects and underlying mechanisms of the water extract of AM (WEAM) in relieving ARCI in D‐gal‐induced aging models, with a specific emphasis on the BDNF/TrkB pathway (Figure 1).

## 2 Materials and Methods

### 2.1 Materials and Reagents

*Allium macrostemon* Bge. (AM, NO.2206149) was obtained from the Neautus Traditional Chinese Medicine Co. Ltd. (Sichuan, CHN). The WEAM is prepared by soaking 40 g powder of AM in 1400 mL of distilled water (w/v) for 60 min, followed by reflux heating extraction for an additional 60 min, and subsequent filtration. This extraction process was repeated three times. The combined filtrate was concentrated and lyophilized using a freeze‐drying device (YTLG‐12‐12A‐80, Shanghai Yetuo Technology, CHN), resulting in a final WEAM yield of 49.88%. Memantine (MEM, No.2206149) was provided by the H. Lundbeck A/S (Denmark); D‐galactose (D‐gal, #HR859W8) and 7,8‐Dihydroxyflavone (7,8‐DHF, #HR9429W1) were sourced from the Herbest Inc. (Baoji, CHN); ANA‐12 (S7745) was obtained from the Selleckchem Inc. (Houston, USA); primary antibodies against BDNF (28205‐1‐AP), TrkB (13129‐1‐AP), BAX (50599‐2‐Ig), β‐Tubulin (10094‐1‐AP), and MAP2 (17490‐1‐AP) were acquired from the Proteintech Group Inc. (Wuhan, CHN); anti‐SYN (ab32127) and BCL‐2 (ab182858) were provided by the Abcam (Cambridge Science Park, UK); anti‐PSD95 (WL05046) was purchased from WanLei Biological Technology Co. Ltd. (Shenyang, CHN); HRP‐conjugated Goat Anti‐Rabbit IgG (GB23303) was provided by the Service Biotech Co. Ltd. (Wuhan, CHN). Standard compounds of macrostemonoside (AFCK2402), sarsasapogenin (AFCG3156), diosgenin (AFCK2452), timosaponin AIII (AF22051605), N‐p‐trans‐coumaroyltyramine (AFCK2401), guanosine (AFCB0855), adenosine (AFCD1351), phenylalanine (AFCE0954), adenine (AFCK1552), arginine (AFCD0552), and valine (AFCD0553) were sourced from Chengdu Alfa Biotechnology Co. Ltd. (Chengdu, CHN).

### 2.2 Animals and Procedure

Kunming mice (equal numbers of males and females, SPF, 6 weeks old, weighing 18–22 g, No. 110324220107338154) were procured from SiPeiFu Biotechnology Co. Ltd. (Beijing, CHN). Animals were housed under controlled temperature conditions (22°C ± 2°C) with 12‐h light/dark and were provided ad libitum access to food. Animal protocols received approval from the Research Ethics Committee of the Institute of Material Medica Integration and Transformation for Brain Disorders, Chengdu University of Traditional Chinese Medicine (No. IBD2022012, November 20, 2022). Animals were assigned to different experimental groups at random: Cont. group, D‐gal group, MEM group (3 mg/kg), WEAM group (0.25, 0.5 and 1.0 g/kg, separately named WEAM‐L, WEAM‐M and WEAM‐H). Each group consisted of 10 animals. All the experimental drugs were dissolved in 0.5% CMC‐Na solution and given intragastric administration. Following oral administration of the respective treatments for 1 h, 500 mg/kg D‐gal (dissolved in saline) was subcutaneously injected daily for 45 days, with the Cont group receiving physiological saline.

### 2.3 Behavior Tests

#### 2.3.1 Y‐Maze Test

Y‐Maze Test (YMT) comprised three arms arranged at 120° angles, with a central triangular area. Mice were put into the central region, and the sequence of arm entries within 5 min was recorded. The spontaneous alternation was utilized to evaluate the learning and memory capabilities of mice (Xu et al. 2022). The spontaneous alternation rate was calculated as follows: spontaneous alternation rate = spontaneous alternation times/(total number of entries into the arm—2) × 100%.

#### 2.3.2 Passive Avoidance Test

Passive Avoidance Test (PAT) was employed to assess the fear memory of mice, following previously established earlier (Zhao et al. 2020). Briefly, mice were acclimatized in a shuttle box for 5 min. Subsequently, Then, an electric shock (0.7 mA) was administered upon entry into the dark compartment. The official test was performed the following day, recording the latency to first enter a dark room within 5 min as step‐through latency, while the frequency of entries into an unilluminated box was recorded as the number of errors.

#### 2.3.3 Morris Water Maze

Morris Water Maze (MWM) was utilized to evaluate the spatial learning and memory abilities of the mice, as previously described (Chen et al. 2023; Zou et al. 2024). In brief, animals underwent training in the MWM apparatus for 5 days, where a hidden platform was used for a directional navigation test. If the mice successfully located the concealed platform within 1 min during daily training, the escape latency was recorded. If unsuccessful, they were guided to the platform and allowed to remain for 10 s, with a maximum escape latency recorded as 60 s. On the sixth day, the hidden platform was removed, and a spatial probe trial was conducted, including the time in the platform zone and the number of crossing the target quadrant, which was automatically recorded by the MWM system (Noldus, Netherlands).

### 2.4 HE, Nissl, and TUNEL Staining

Mice brain tissue was fixed in a universal tissue fixative for 72 h. Dehydration and subsequent paraffin embedding of brain tissue were operated in adherence to standard protocol using an automatic dehydrator (Laica). The brain tissue was sectioned to 4‐μm slices along the coronal plane, mounted on slides, and dried at 42°C. For HE (G1005, Servicebio, CHN) and Nissl staining (C0117, Beyotime Biotechnology, CHN), slices underwent deparaffinization and staining following standard operating procedures. For TUNEL staining (AK80256, Elabscience, CHN), slices were strictly processed according to the manufacturer's instructions, involving dewaxing, protease K treatment, and incubation with Terminal deoxynucleotidyl transferase (TdT) and fluorescein‐dUTP for 1 h. Finally, slices were sealed with an antifade mounting medium with DAPI (S2110, Solarbio, CHN) and observed under a microscope (Sunny, CHN).

### 2.5 Reactive Oxygen Species

Brain tissues were collected after cold saline transcardial perfusion after being anesthetized with pentobarbital. A single cell suspension was prepared, loaded with a DCFH‐DA probe according to the manufacturer's instructions, and analyzed via flow cytometry (S0033S, Beyotime Biotechnology, CHN). Results were processed using FlowJo software to generate the fluorescence histogram and determine the percentage of positive cells.

### 2.6 Kit Detection

Superoxide dismutase (SOD) activity and malondialdehyde (MDA) levels in the brain tissue of mice were meticulously assessed, following the operational steps outlined in SOD (A001‐3‐2, Nanjing Jiancheng Bioengineering Institute, CHN) and MDA assay kits (S0131S, Beyotime Biotechnology, CHN).

### 2.7 Cell Culture

SH‐SY5Y cells were obtained from the Chinese Academy of Sciences (Shanghai, China) and hatched in DMEM medium containing 10% fetal bovine serum (CellMax, CHN) and 1% streptomycin/ penicillin in a 5% CO_2_ incubator at 37°C. For efficacy testing, cells were seeded into 96‐well plates at a density of 5 × 10^3^ cells/hole. 12 h post‐seeding, cells were stimulated with 40 mg/mL D‐gal in DMEM for an additional 12 h. Subsequently, cells were treated with ANA‐12 (10 μM) or 7,8‐DHF (5 μM) for 4 h, followed by administration of WEAM (5 μg/mL) for 8 h. After treatment, 10% CCK8 was placed into each hole and hatched for 2 h at 37°C. Finally, cell viability was detected using an enzyme‐labeling instrument at 450 nm. This test was independently repeated at least three times.

### 2.8 Hoechst Staining and Synaptic Staining

For Hoechst staining, cells were fixed with 4% paraformaldehyde for 30 min at 25°C, followed by incubation with Hoechst 33258 for 10 min, and then visualized and captured by a fluorescent microscope. For synaptic staining, the fixation procedure was similar to that of Hoechst staining. Fixed SH‐SY5Y cells were blocked with goat serum for 30 min at 25°C, hatched with MAP2 antibody (1:200) overnight at 4°C, and subsequently treated with a secondary antibody for 1 h at 25°C. Finally, an anti‐fluorescence quencher containing DAPI was applied to seal the slices, and then immunofluorescence images were obtained by a fluorescent microscope.

### 2.9 Western Blotting

Mice brain or SH‐SYSY cells were homogenized in lysis buffer and disrupted using a cell ultrasonic crusher. The protein concentration was adjusted to 4 μg/uL for brain tissues or 1 μg/uL for cells using PBS and mixed with loading buffer. Target proteins were separated via SDS‐PAGE and subsequently transferred to PVDF membranes. Subsequently, the membranes were blocked, incubated with the corresponding primary antibodies at 4°C overnight, and subjected to 1‐h incubation with secondary antibody at 25°C. Finally, the membranes were visualized and imaged using an e‐bolt advice and analyzed using Image J software.

### 2.10 Mass Spectrometry Analysis of WEAM

The freeze‐dried powder of WEAM was dissolved in methanol via ultrasonic treatment, centrifugated for 10 min at 3500 rpm/min, and filtered using a 0.22 μm millipore filter to prepare the sample solution for HPLC‐MS/MS. A quadrupole‐electrostatic field orbitrap high‐resolution mass spectrometer (Thermo Fisher Scientific, USA) was employed to analyze the compounds in WEAM. The sample was loaded onto an Agilent Eclipse Plus C18 RRHD (3.0 × 150 mm, 1.8 μm, Agilent, CA, USA) at 40°C. The ionization source was performed in both positive and negative ion modes at an electrospray voltage of 3.5 and 3.0 KV, respectively, with a temperature of 320°C. The heating temperatures of auxiliary gas were set at 300°C. The mobile phase consisted of H_2_O (containing 0.1% formic acid, A) and acetonitrile (B). The gradient elution program was as follows: 0–4 min, 5% (B); 4–51 min, 5%—100% (B); 51–56 min, 100% (B). Injection volume: 5 μL; flow rate: 0.2 mL/min; and the scan range: 100–1000 m/z.

### 2.11 Statistical Analysis

The results were presented as mean ± standard error of the mean (SEM). ANOVA was utilized to analyze the escape latency in MWM, while one‐way ANOVA was applied for comparisons of all other data. Differences between the two groups were analyzed with Student's *t*‐test, with *p* < 0.05 recognized as statistically significant.

## 3 Results

### 3.1 WEAM Alleviated Cognitive Deficits and Neuropathologic Lesions in D‐Gal‐Induced Aging Mice

**FIGURE 2:**
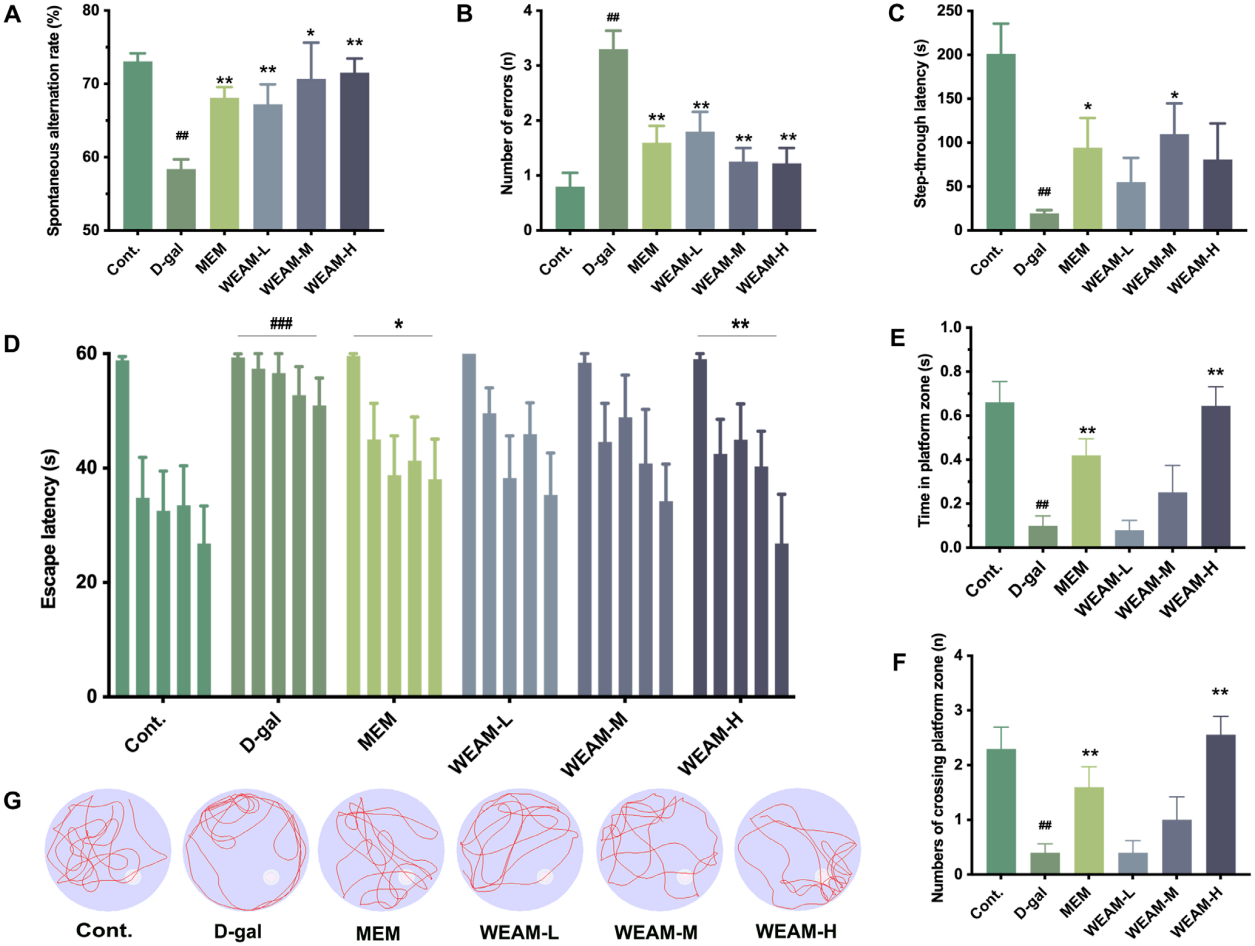
WEAM alleviated cognitive deficits in D‐gal‐induced aging mice. (A) The spontaneous alternation rate within 5 min in the Y‐Maze test (*n* = 10). (B) Number of errors and (C) step‐through latency within 5 min in the PAT test (*n* = 10). (D)The escape latency, (E)swimming time in the platform zone, and (F) the numbers of crossing the platform zone, and (G) representative swimming traces of mice in the MWM test (*n* = 10). All data are presented as mean ± SEM. ^#^ *p* < 0.05 or ^##^ *p <* 0.01 versus cont. group; *p < 0.05 or **p < 0.01 versus the D‐gal mice.

**FIGURE 3:**
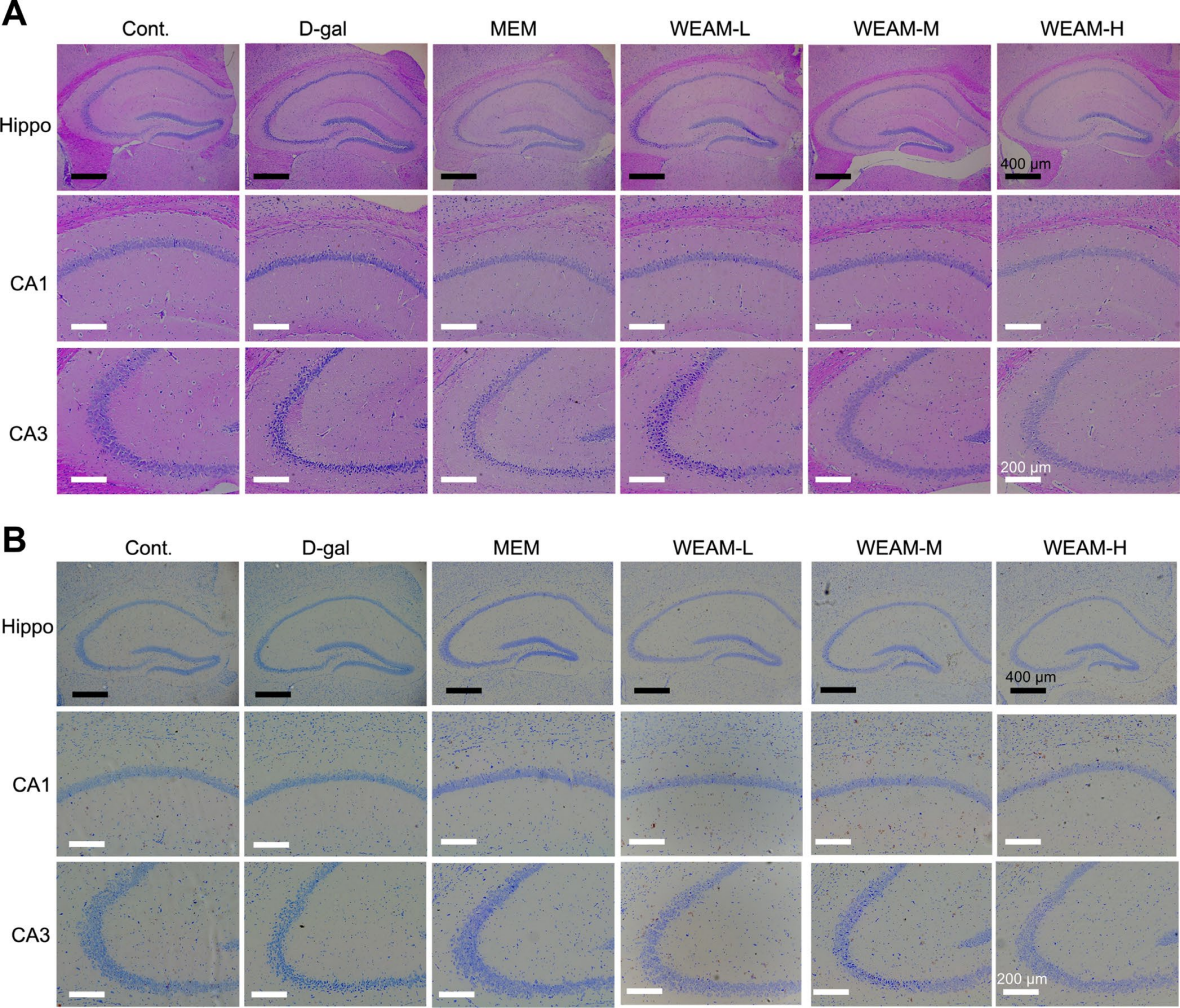
WEAM alleviated neuropathologic lesions in D‐gal‐induced aging mice. (A) The representative images of HE staining and (B) Nissl staining in the hippocampal CA1 and CA3 regions (*n* = 3–4), Scale bars = 200 or 400 μm.

The effects of sex‐imparted differences on cognitive function were first evaluated, and our data showed no significant differences between male and female mice within each group (Figure S1). Therefore, male and female mice in each group were combined in the following study. In the Y‐Maze test, the spontaneous alternation rate in aging mice was lower than that observed in Cont mice, while WEAM administration increased this rate dose‐related (Figure 2A; *p* < 0.05 or *p <* 0.01). In the PAT test, D‐gal mice exhibited more errors and a reduced step‐through latency compared to Cont mice, as anticipated, WEAM reversed these alterations in a dose‐associated fashion (Figure 2B,C; *p <* 0.05 or *p <* 0.01). In the MWM test, as seen in Figure 2D, escape latency decreased over time [F (4, 150) = 13.74, *p* < 0.0001], with D‐gal mice demonstrating longer escape latencies than Cont mice [F (1, 50) = 25.69, *p* < 0.0001]. Notably, escape latencies were reduced following treatment with both MEM [F (1, 50) = 5.93, *p* = 0.0185] and WEAM‐H [F (1, 50) = 9.24, *p* = 0.0038]. Furthermore, the number of crossing the platform zone and the time in this zone were diminished in D‐gal mice compared to Cont mice; however, these deficits were substantially ameliorated by MEM and WEAM‐H (Figure 2E–G; *p <* 0.01). ARCI is associated with neuropathologic lesions in hippocampal neurons (Nyberg and Pudas 2019). Histological examination via HE staining revealed increased intercellular spacing, disorganized cellular arrangement, and neuronal loss in the CA1 and CA3 of the hippocampus in D‐gal mice compared with Cont mice. Conversely, these neuropathologic impairments were ameliorated in WEAM‐treated mice (Figure 3A). Additionally, the number of Nissl bodies of mice in the WEAM group was greater than that in the D‐gal group (Figure 3B). These findings suggest WEAM alleviates cognitive dysfunction and neuropathological damage induced by D‐gal.

### 3.2 WEAM Inhibited Hippocampal Neuronal Apoptosis in D‐Gal Aging Mice

**FIGURE 4:**
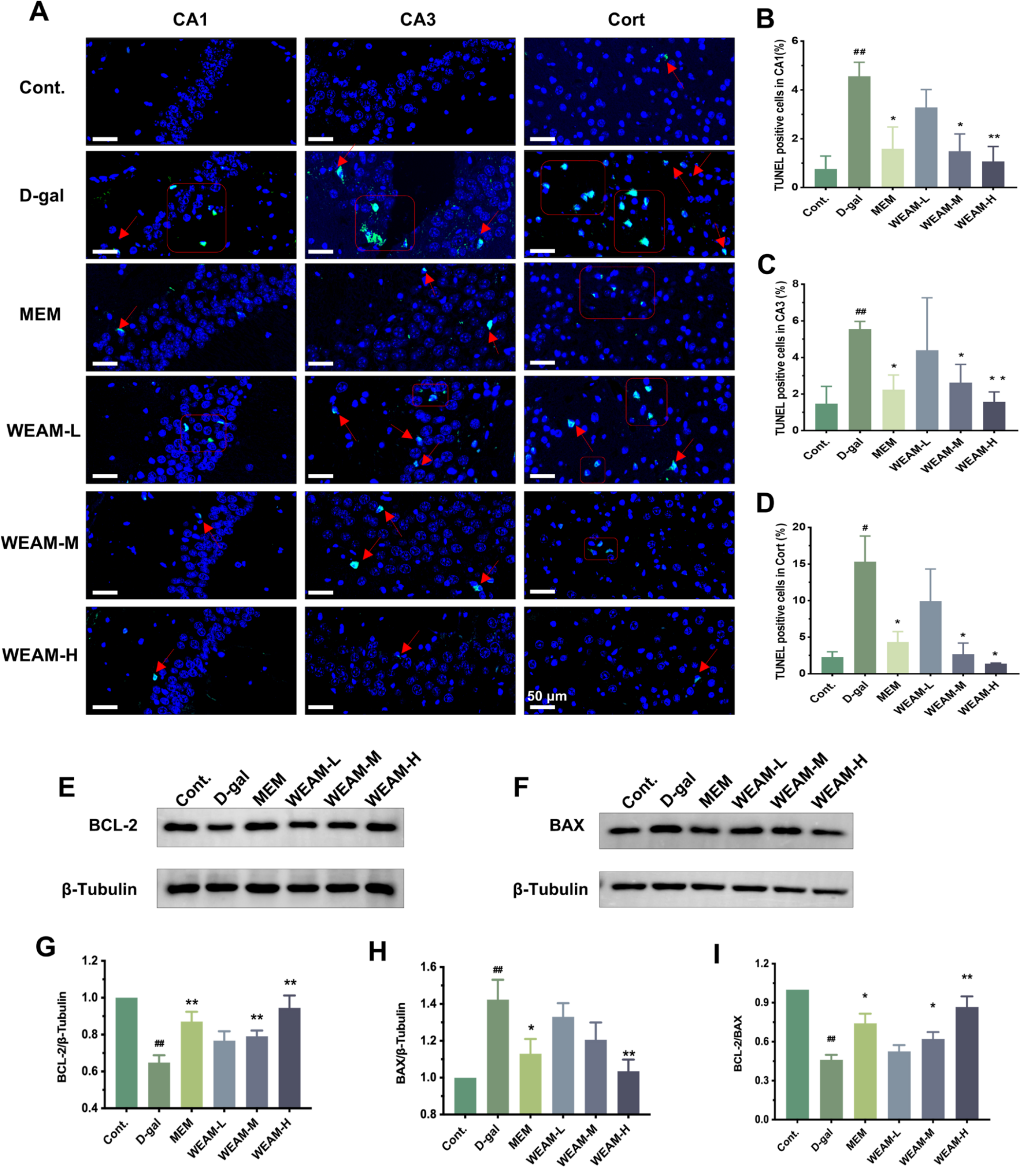
WEAM inhibited hippocampal neuronal apoptosis in D‐gal aging mice. (A) Representative pictures of TUNEL^+^ staining in the hippocampal and cortex (Cort) regions, the red arrows represent apoptotic cells, Scale bars = 50 μm, *n* = 3. The percentage of TUNEL^+^ cells in the hippocampal CA1 (B), CA3 (C), and Cort (D) regions, *n* = 3. (E–I) Western blot and quantitative analyses of the BCL‐2 and BAX protein expression (*n* = 3). All data are presented as mean ± SEM. ^#^ *p <* 0.05 or ^##^ *p <* 0.01 versus the cont. group; *p < 0.05 or **p < 0.01 versus the D‐gal group.

Excessive apoptosis is a critical mechanism underlying neuronal loss in the aging hippocampus. TUNEL staining results indicated that the number of TUNEL^+^ cells in the CA1, CA3 of hippocampal, and cortical regions of D‐gal mice was higher than that in Cont mice (*p <* 0.05 or *p <* 0.01), whereas WEAM treatment reduced the number of TUNEL^+^ cells in those regions (Figure 4A–D; *p <* 0.05 or *p <* 0.01). Similarly, the expression of BCL‐2 was down‐regulated in D‐gal mice compared to Cont mice, while the BAX level was elevated, resulting in a decreased BCL‐2/Bax ratio (Figure 4E–I; *p <* 0.05 or *p <* 0.01). As expected, WEAM treatment reversed these alterations in the expression of apoptosis‐related proteins (Figure 4E–I; *p <* 0.05 or *p <* 0.01). These findings indicate that WEAM effectively inhibits neuronal apoptosis in aging mice.

### 3.3 WEAM Alleviated Oxidative Stress Induced by D‐Gal in Mice

**FIGURE 5:**
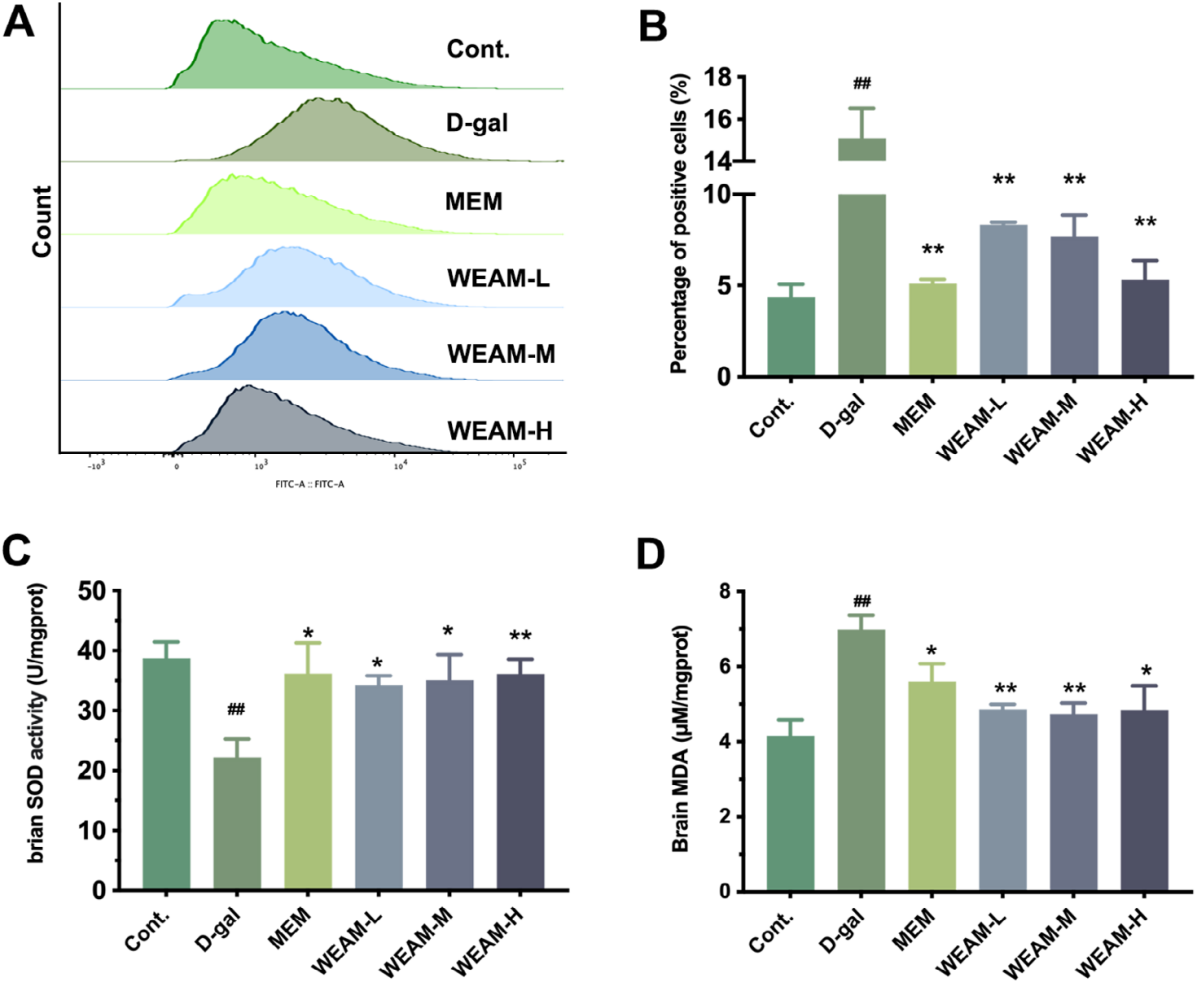
WEAM alleviated oxidative stress induced by D‐gal in mice. (A, B) Representative images and quantification of the ROS fluorescence intensity by flow cytometry (*n* = 3). (C) The activity of SOD in brain tissue of mice (*n* = 5). (D) The content of MDA in brain tissue of mice (*n* = 5). All data are presented as mean ± SEM. ^##^ *p <* 0.01 versus the cont. group, *p < 0.05 or **p < 0.01versus the D‐gal group.

Accumulated oxidative stress is a fundamental mechanism contributing to ARCI and the onset of neurodegenerative diseases (Ionescu‐Tucker and Cotman 2021). Prolonged exposure to D‐gal can induce brain aging and cognitive impairment through the enhancement of oxidative stress (Shwe et al. 2018). Our results demonstrated that D‐gal caused ROS release (Figure 5A,B; *p <* 0.01), decreased SOD activity (Figure 5C; *p <* 0.01), and increased MDA levels (Figure 5D; *p <* 0.01) compared to Cont mice. However, these oxidative stress‐related changes were ameliorated by WEAM treatment (Figure 5A–D; *p <* 0.05 or *p <* 0.01), suggesting WEAM mitigates oxidative stress impairment in the brain of D‐gal mice.

### 3.4 WEAM Enhanced Neuronal Plasticity Through BDNF/TrkB Pathways in D‐Gal Mice

**FIGURE 6:**
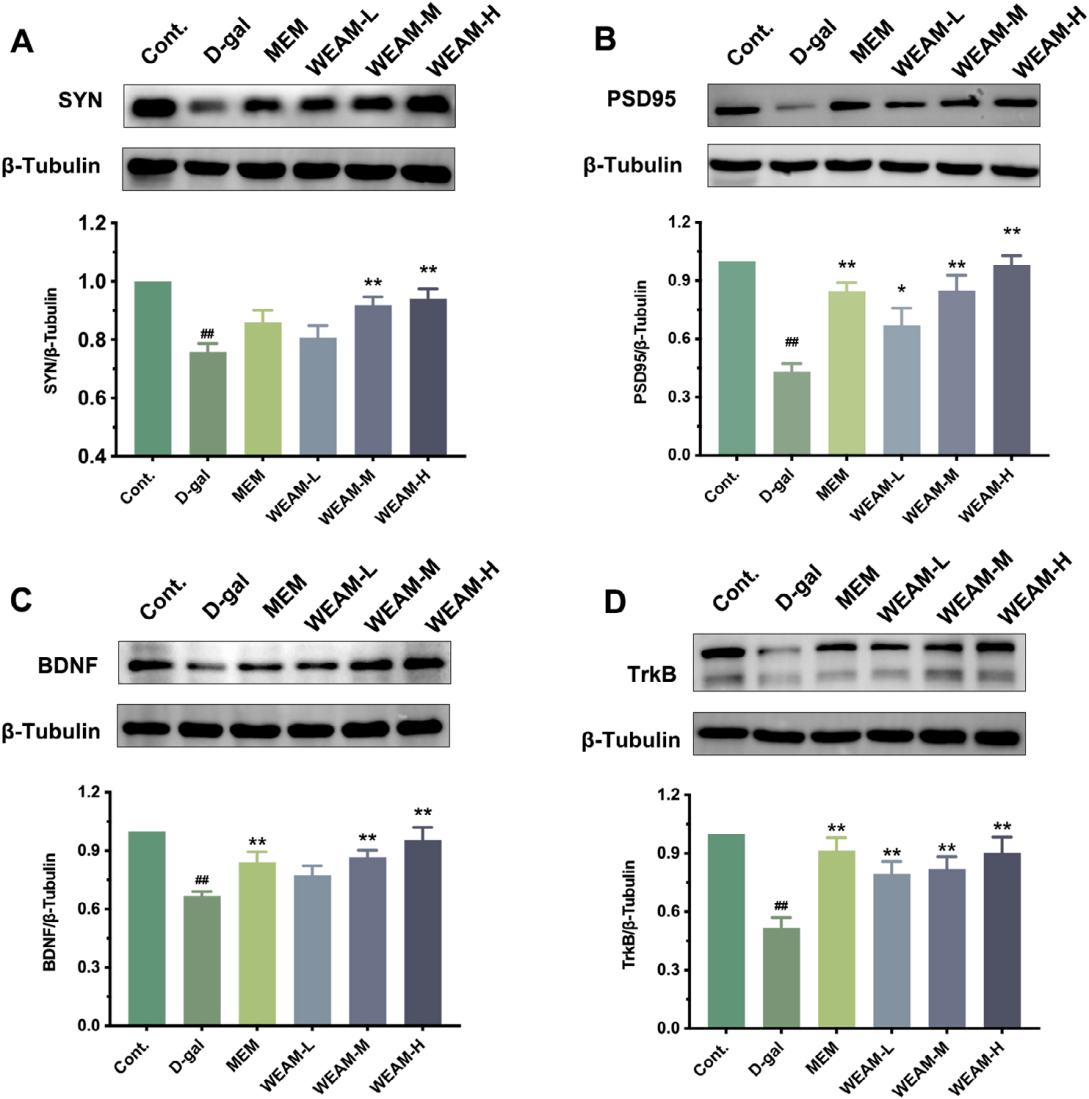
WEAM enhanced neuronal plasticity through BDNF/TrkB pathways in vivo. Representative western blot bands and quantitative analyses of the SYN (A), PSD95 (B), BDNF (C), and TrkB (D) protein (*n* = 3). All data are presented as mean ± SEM, ^##^ *p <* 0.01 versus the cont. group, *p < 0.05 or **p < 0.01 versus the D‐gal group.

In addition to neuronal apoptosis and oxidative damage, the aging process also involves changes in synaptic plasticity (Plascencia‐Villa and Perry 2023). Adequate BDNF/TrkB signaling is critical for neuronal survival, oxidative damage, and maintenance of synaptic plasticity (Min et al. 2023; Wang et al. 2024). Presynaptic and postsynaptic proteins, such as SYN and PSD95, are recognized as markers of synaptic plasticity (Pan et al. 2023; Zhou et al. 2023). The SYN and PSD95 expression levels in D‐gal‐induced aging mice were lower than in Cont mice (Figure 6A,B; *p <* 0.01). However, following WEAM administration, the levels of these proteins increased dose‐dependent (Figure 6A,B; *p <* 0.05 or *p <* 0.01). The BDNF/TrkB signaling was inhibited in aging mice compared to Cont mice, as evidenced by decreased expression of associated proteins, including BDNF and TrkB (Figure 6C,D; *p <* 0.01). Nevertheless, WEAM treatment restored the expression of BDNF and TrkB proteins (Figure 6C,D; *p <* 0.01). These findings hint that WEAM enhances synaptic plasticity by activating the BDNF/TrkB pathway in aging mice.

### 3.5 7,8‐Dihydroxyflavone Enhanced the Neuroprotective Effects of WEAM In Vitro

**FIGURE 7:**
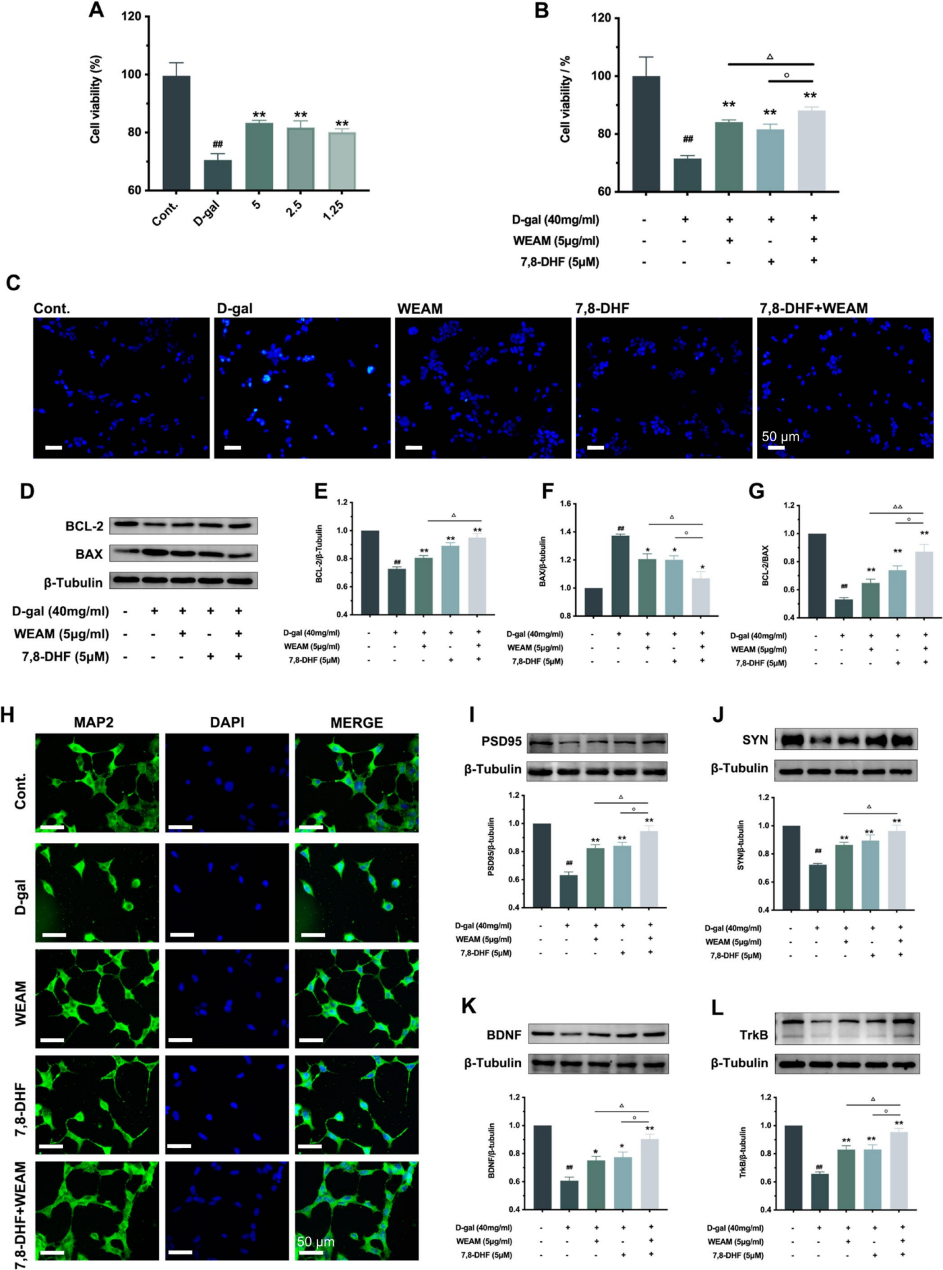
7,8‐DHF enhanced the neuroprotective effects of WEAM in vitro. (A) The effect of different concentrations of WEAM in cell viability of D‐gal‐induced SH‐SY5Y cells (*n* = 5); (B) The synergistic effect of WEAM and 7,8‐DHF on cell viability of D‐gal‐induced SH‐SY5Y cells (*n* = 5); (C) Representative images of Hoechst 33258 stain (*n* = 3), Scale bars = 50 μm; (D–G) representative western blots and quantitative analysis of BCL‐2 and BAX (*n* = 3); (H) Representative images of MAP2 fluorescence staining (*n* = 3), Scale bars = 50 μm; (I–L) western blot and quantitative analysis of PSD95, SYN, BDNF and TrkB in SH‐SY5Y cells (*n* = 3). All data are presented as mean ± SEM, ^##^ *p <* 0.01 versus the cont. group, *p < 0.05 or **p < 0.01 versus the D‐gal group, ^△^ *p <* 0.05 versus the WEAM group, ^○^ *p <* 0.05 versus the 7,8‐DHF group.

7,8‐dihydroxyflavone (7,8‐DHF) is a TrkB activator miming BDNF (Chen et al. 2018). D‐gal‐treated SH‐SY5Y cells were established in vitro. Our data indicated that WEAM mitigated cell damage induced by D‐gal in a dose‐dependent manner (Figure 7A,B; *p <* 0.01) and inhibited neuronal apoptosis in vitro, as evidenced by decreased Hoechst^+^ expression (Figure 7C), increased BCL‐2 expression, and decreased BAX levels (Figure 7D–G; *p <* 0.05 or *p <* 0.01). Moreover, WEAM protected synapsis, as demonstrated by the alleviation of morphological damage in the synaptic marker MAP2 staining (Figure 7H) and the up‐regulation of SYN and PSD95 levels (Figure 7I,J; *p <* 0.01). Furthermore, WEAM increased the expression of BDNF and TrkB (Figure 7K,L; *p <* 0.05 or *p <* 0.01). Notably, these effects of WEAM were enhanced in the presence of 7,8‐DHF (Figure 7B–L; *p <* 0.05 or *p <* 0.01).

### 3.6 ANA‐12 Reversed the Neuroprotective Effect of WEAM In Vitro

**FIGURE 8:**
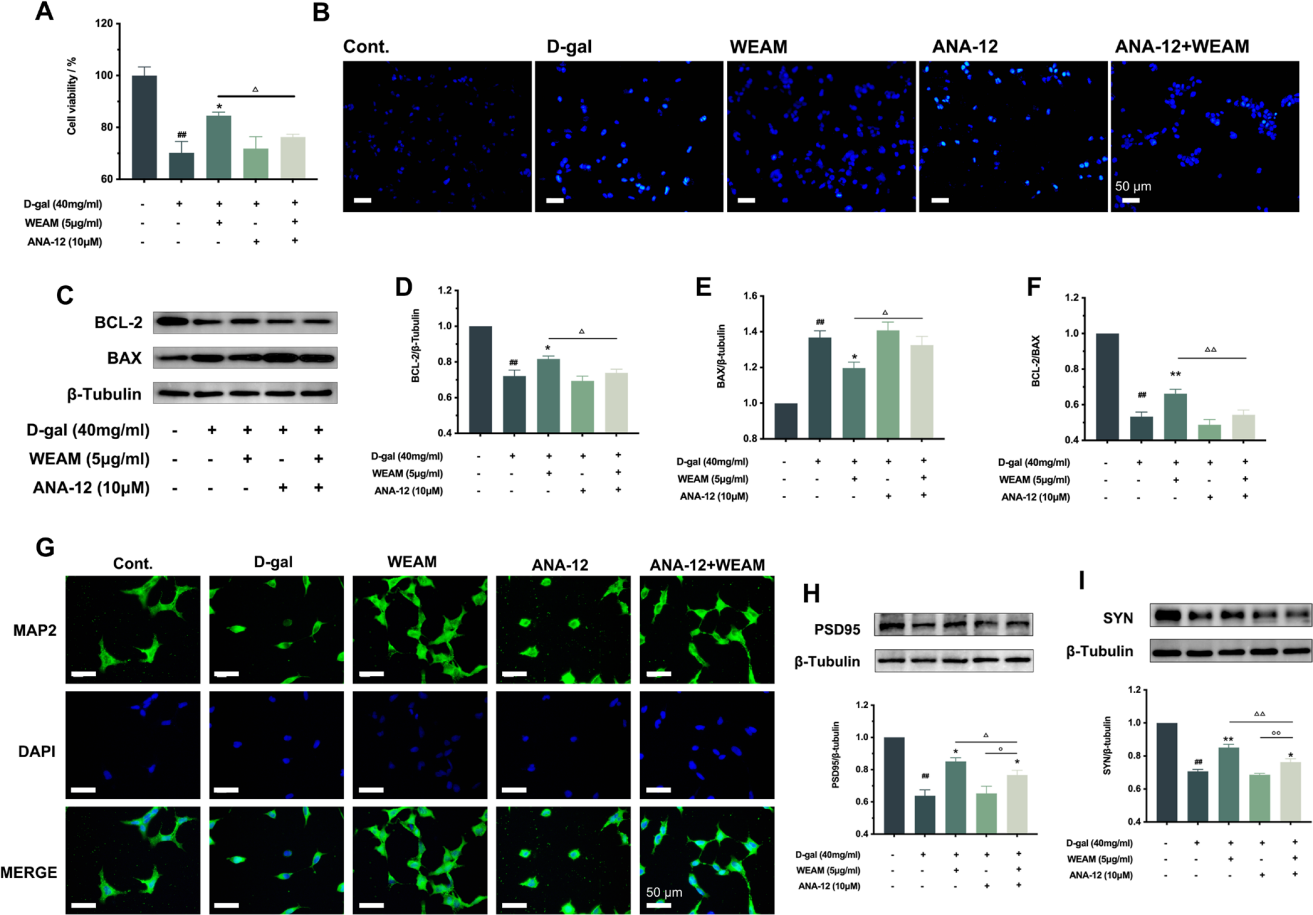
ANA‐12 reversed the neuroprotective effect of WEAM in vitro. (A) The effect of WEAM in cell viability of D‐gal‐induced SH‐SY5Y cells after ANA‐12 was added (*n* = 5); (B) Representative images of Hoechst 33258 stain (*n* = 3), Scale bars = 50 μm; (C–F) Representative western blots and quantitative analysis of BCL‐2 and BAX (*n* = 3); (G) Representative images of MAP2 fluorescence staining (*n* = 3), Scale bars = 50 μm; (H–I) western blot and quantitative analysis PSD95 and SYN in SH‐SY5Y cells (*n* = 3). All data are presented as mean ± SEM, three independent experiments, ^##^ *p <* 0.01 versus the cont. group, *p < 0.05 or **p < 0.01 versus the D‐gal group, ^△^ *p* < 0.05 or ^△△^ *p <* 0.01 versus the WEAM group, ^○^ *p* < 0.05 or ^○○^ *p <* 0.01 versus the ANA‐12 group.

To further elucidate whether the protective effects of WEAM are dependent on TrkB, ANA‐12 (a TrkB inhibitor) was utilized in vitro. The enhancement of WEAM on cell viability in vitro was partially reversed by ANA‐12 (Figure 8A; *p <* 0.05). As expected, the anti‐apoptosis effects of WEAM were also reversed by ANA‐12 (Figure 8B–F; *p <* 0.05 or *p <* 0.01). Additionally, synaptic damage was inhibited by ANA‐12 compared to the WEAM administration group (Figure 8G). Furthermore, the increased effects of WEAM on synaptic plasticity (as indicated by the expression of SYN and PSD95) were also reversed by ANA‐12 (Figure 8H,I; *p <* 0.05 or *p <* 0.01). These results further suggest that WEAM protected against D‐gal‐caused neuronal damage by activating the BDNF/TrkB pathway.

### 3.7 Eleven Compounds Were Identified in WEAM

**FIGURE 9:**
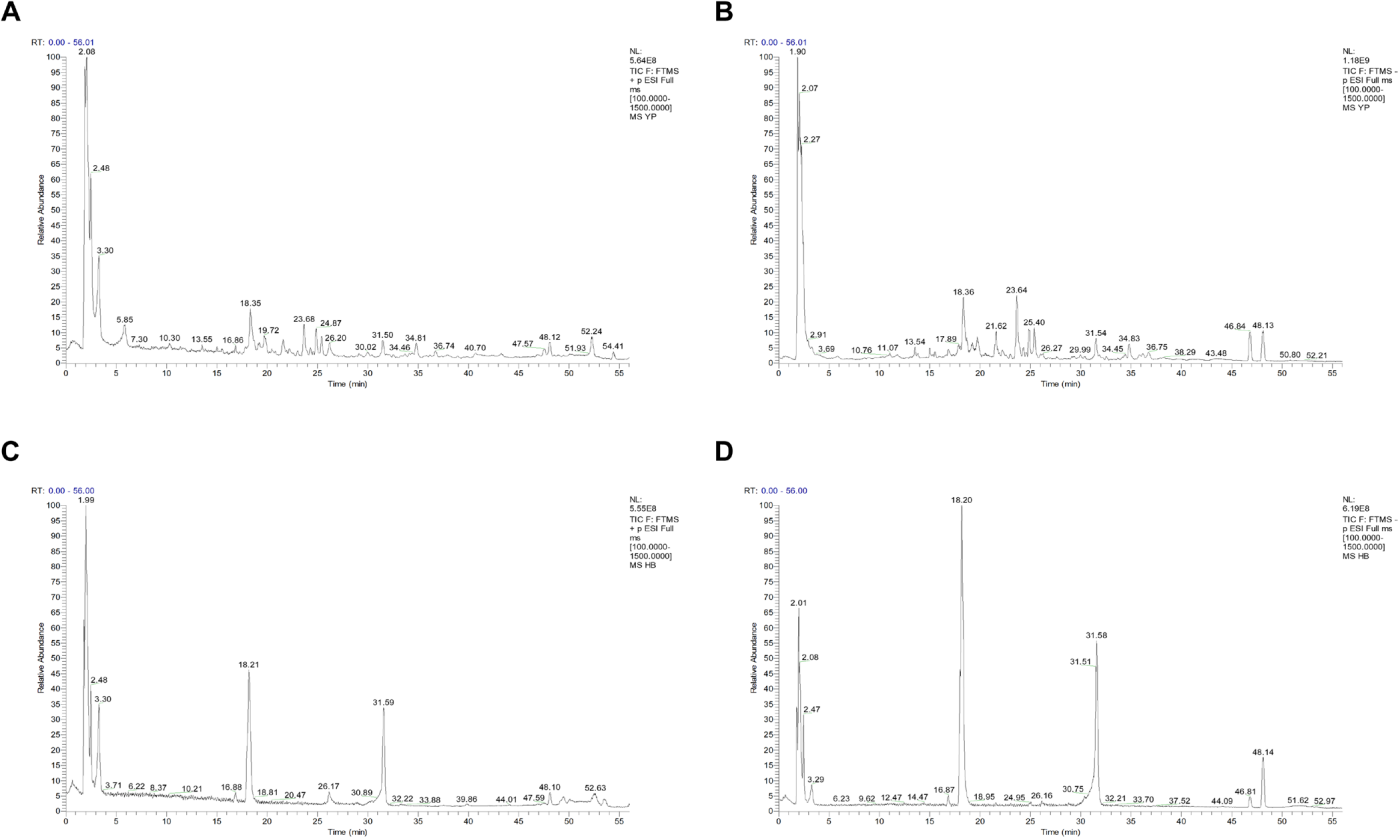
HPLC‐MS/MS showed the ingredients in the WEAM. (A, B) Total ion current scan chromatogram of positive ions and negative ions of WEAM. (C, D) Total ion current scan chromatogram of positive ions of standard compounds and negative ions of standard compounds.

To explore the components of WEAM, HPLC‐MS/MS was employed for qualitative analysis. The total ion chromatograms of both positive and negative ion modes are presented in Figure 9A,B. Eleven ingredients were identified and confirmed, as detailed in Table 1. The total ion chromatograms for these 11 standard compounds are depicted in Figure 9C,D, with their respective contents in WEAM summarized in Table 1.

**TABLE 1:** The content of compounds in WEAM.

Chemical compound	Formula	Rt (min)	MS^1^	MS/MS fragment	Content (μg/g)	Ref.
Arginine	C_6_H_14_N_4_O_2_	1.81	175.119 [M + H]^+^	158.09, 130.10, 116.07, 112.09	6.03	Sun et al. (2022)
Valine	C_5_H_11_NO_2_	1.98	118.086 [M + H]^+^	72.08, 57.06, 55.05	25.60	Sun et al. (2022)
Adenosine	C_10_H_13_N_5_O_4_	2.00	268.104 [M + H]^+^	136.06, 119.04	77.13	Gao et al. (2023)
Guanosine	C_10_H_13_N_5_O_5_	2.02	284.099 [M + H]^+^	152.06, 135.03, 110.04	7.52	Gao et al. (2023)
Phenylalanine	C_9_H_11_NO_2_	2.16	166.086 [M + H]^+^	120.08,103.05	657.20	Gao et al. (2023)
Adenine	C_5_H_5_N_5_	2.35	136.062 [M + H]^+^	119.04, 94.04, 67.03	106.36	Sun et al. (2022)
N‐p‐trans‐Coumaroyltyramine	C_17_H_17_NO_3_	18.21	284.294 [M + H]^+^	147.04, 121.06, 103.05	2.01	Sun et al. (2022)
Macrostemonoside	C_45_H_76_O_19_	18.34	920.518 [M + H]^+^	741.44, 579.39, 417.34	121.76	Sun et al. (2022)
Timosaponin AIII	C_39_H_64_O_13_	18.37	741.440 [M + H]^+^	579.39, 435.27, 417.34, 273.22	39.17	Sun et al. (2022)
Sarsasapogenin	C_27_H_44_O_3_	31.53	417.335 [M + H]^+^	417.33, 273.22, 255.21	51.53	Dai et al. (2023)
Diosgenin	C_27_H_42_O_3_	31.59	415.319 [M + H]^+^	415.32, 271.20, 253.19	15.04	Zeng et al. (2024)

## 4 Discussion

*Allium macrostemon* Bge. a common vegetable and culinary spice, has exhibited beneficial effects on various diseases, including atherosclerosis (Liu et al. 2022), obesity (Kim, Lee, and Kim 2023), pain (Yang et al. 2021), and asthma (Wu et al. 2023). However, its potential to alleviate ARCI remains unclear. Our findings indicate that WEAM attenuates D‐gal‐induced cognitive impairment widely utilized in ARCI studies (Ullah et al. 2020). Aging‐induced neuronal damage serves as the structural basis of cognitive decline (Dahan, Rampon, and Florian 2020; Landfield 1988). The inhibitory effect of WEAM on neuronal damage confirms its neuroprotective effects. The disruption of brain REDOX balance causes cellular damage and impairs synaptic plasticity during aging, leading to neuronal death and cognitive decline (Plascencia‐Villa and Perry 2023; Verdú et al. 2023). In this study, WEAM reduced oxidative damage and apoptosis in the brains of D‐gal‐induced model mice, confirming its antioxidant and anti‐apoptotic effects again (Gao et al. 2023; Wu et al. 2020). The impairment of synaptic plasticity is closely related to age‐related neurological diseases, such as Alzheimer's disease (AD), Parkinson's disease (PD), and ARCI (Shadfar et al. 2023). Synaptophysin (SYN) is a critical protein involved in various processes, including synaptic biogenesis, formation, vesicle protein sorting, endocytosis, and exocytosis, all of which are essential for synaptic plasticity (Mishra and Thakur 2024). A reduction in PSD95 levels constrains synaptic transmission efficiency, a phenomenon long recognized as indicative of cognitive impairment (Tian et al. 2024). The impact of WEAM on synaptic plasticity in aging mice was confirmed by quantifying SYN and PSD95 protein expression levels, which first demonstrated that AM can influence synaptic plasticity.

As a neurotrophic factor, BDNF is particularly relevant to cognitive function (Wang, Kavalali, and Monteggia 2022; Tian et al. 2024). BDNF interacts with TrkB, regulating the synthesis of localized synaptic proteins, thereby preserving synaptic structure and enhancing synaptic plasticity (Wang, Kavalali, and Monteggia 2022; Leal et al. 2015). Current evidence indicates that the levels of BDNF and TrkB are negatively correlated with aging and are closely involved in ARCI (Numakawa and Odaka 2022; Oh, Lewis, and Sibille 2016). Our current study confirmed that WEAM alleviated BDNF/TrkB signaling inhibition in aging mice (Hong et al. 2023; Zheng et al. 2023). TrkB receptor agonists exhibit reliable neuroprotective effects and have been shown to alleviate various neuronal injuries and cognitive decline (Wang et al. 2024; Jang et al. 2010). Recent evidence indicates that TrkBs agonists, such as LM22A‐4 and 7,8‐DHF, benefit myocardial function after myocardial ischemia with mechanisms like that described in the brain (Cannavo et al. 2023). Conversely, TrkB inhibitors, such as ANA‐12, impair hippocampus‐dependent learning and memory (Sun et al. 2023). Our data showed the neuroprotective effect of WEAM was partially attenuated by ANA‐12 but potentiated by 7,8‐DHF. These findings further substantiate that the BDNF/TrkB pathway mediates the neuroprotective effects of WEAM. Additionally, our data also support the critical role of the BDNF/TrkB pathway in age‐related cognitive dysfunction (Numakawa and Odaka 2022; Wang et al. 2024).

The active compounds identified in WEAM provide a strong material foundation for their potential efficacy in attenuating ARCI. Consistent with previous literature (Gao et al. 2023; He et al. 2018; Wu et al. 2023), WEAM primarily contains steroids, steroidal saponins, nitrogen‐containing components, phenylpropanoids, and amino acids. Studies have shown that saponins are significant neuroprotective agents, with ginsenosides, astragaloside, sarsasapogenin, and timosaponin being among the most studied (Abduljawad et al. 2022; Yang et al. 2023; Yao, Zhang, and Wang 2023). Sarsasapogenin has been shown to alleviate diabetic encephalopathy (Zhang et al. 2021). Timosaponin AIII can ameliorate cognitive impairment by inhibiting acetylcholinesterase (Lee, Jung, and Kim 2009). Diosgenin has been shown to mitigate brain aging by reducing oxidative stress and inflammation (Qi et al. 2019). These may be key active components of WEAM for alleviating oxidative damage. Increasing levels of L‐arginine and Guanosine exert beneficial neuroprotective activities in various models (He et al. 2024; Lanznaster et al. 2016). Additionally, the protective effect of diosgenin, sarsasapogenin, and macrostemonoside on cardiovascular lesions may also be the key to the upregulation of the BDNF/TrKB signaling pathway by WEAM (Wu et al. 2024; Zhai et al. 2023; Wang and Wang 2022).

Our study still has some limitations. First, although the DCFH‐DA probe was employed to detect ROS production in this work, the specificity of this method may be affected by some other interference factors like iron (Kalyanaraman et al. 2012). Furthermore, the key active ingredient in WEAM that activates the BDNF/TrkB signaling pathway remains to be determined in future studies.

## 5 Conclusion

In conclusion, the work first established that WEAM ameliorated ARCI, with its mechanisms primarily involving the attenuation of oxidative stress and synaptic damage through activating the BDNF/TrkB pathway. These results hint that *Allium macrostemon* Bge. may serve as a potential culinary spice for improving aging and ARCI.

## Author Contributions

**Ruilin Sheng:** data curation (equal), investigation (equal), methodology (equal), writing – original draft (equal). **Meihuan Zhao:** investigation (equal), methodology (equal), writing – review and editing (equal). **Keting Pu:** data curation (equal), methodology (equal). **Yongtao Zhou:** data curation (equal), methodology (equal). **Li Zeng:** data curation (equal), methodology (equal). **Yuanyuan Chen:** data curation (equal), methodology (equal). **Ping Wang:** methodology (equal), writing – review and editing (equal). **Xiao Liu:** methodology (equal), writing – review and editing (equal). **Shijun Xu:** conceptualization (equal), funding acquisition (equal), resources (equal), supervision (equal), writing – review and editing (equal).

## Ethics Statement

The animal experiments conducted in this study were performed in accordance with the guidelines set forth by the Research Ethics Committee of the Institute of Material Medica Integration and Transformation for Brain Disorders at Chengdu University of Traditional Chinese Medicine (Approval No. IBD2022012, dated November 20, 2022).

## Conflicts of Interest

The authors declare no conflicts of interest.

## Supporting information


Figure S1.

## Data Availability

The data that support the findings of this study are available on request from the corresponding author.
