# Supplementary material for: Allium Macrostemon Bge. Attenuates the Cognitive Decline of Aging Mice by Enhancing BDNF/TrkB Pathway
Source: Food Sci Nutr. 2025 Feb 28;13(3):e70010. doi: 10.1002/fsn3.70010 (PMC11868736; doi:10.1002/fsn3.70010)

**Supplementary materials**

**Figure S1 Effects of sex differences within each group on cognitive function in mice.**

(A) The spontaneous alternation rate within 5 min in the Y-Maze test (n=5). (B) Escape latency and (C) number of errors within 5 min in the PAT test (n=5). (D)The escape latency on the fifth day of the directional navigation experiment in the MWM test (n=5). The ns represents not statistically significant.


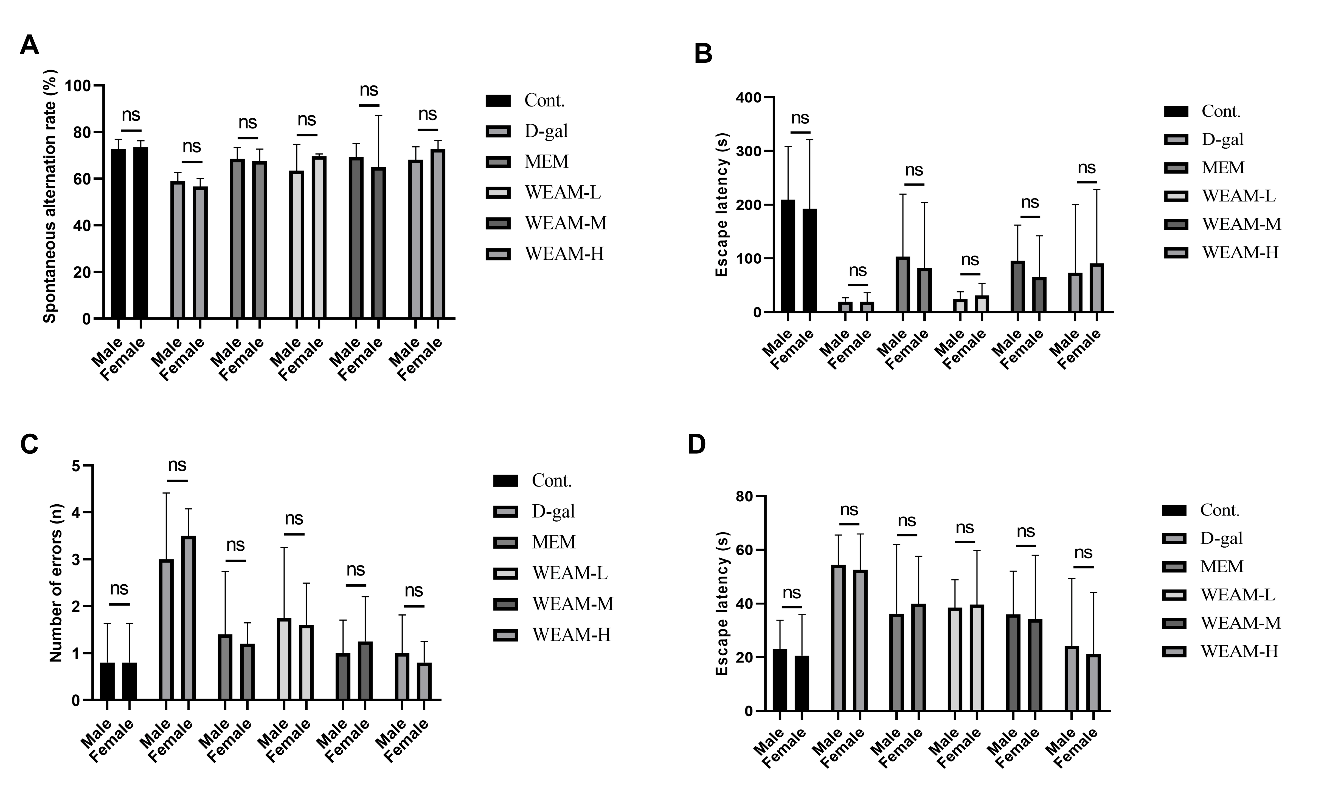

Supplement: Supplementary file 1 — Figure S1. [file FSN3-13-e70010-s001.docx]
